# Cellulose-Based Wet Wipes Combining Essential Oils and Sodium Hypochlorite: A Sustainable Strategy for Bacterial Control

**DOI:** 10.3390/polym18192406

**Published:** 2026-10-02

**Authors:** Zorica Tomičić, Milorad Miljić, Ljubiša Šarić, Dragana Tomanić, Miloš Pelić, Milica Nićetin, Ružica Tomičić

**Affiliations:** 1 Institute of Food Technology in Novi Sad, University of Novi Sad, Bulevar cara Lazara 1, 21000 Novi Sad, Serbia; milorad.miljic@fins.uns.ac.rs (M.M.); ljubisa.saric@fins.uns.ac.rs (L.Š.); dragana.tomanic@fins.uns.ac.rs (D.T.); milos.pelic@fins.uns.ac.rs (M.P.); 2 Faculty of Technology, University of Novi Sad, Bulevar cara Lazara 1, 21000 Novi Sad, Serbia; milican@uns.ac.rs (M.N.); ruzica.tomicic@uns.ac.rs (R.T.)

**Keywords:** cellulose-based materials, sodium hypochlorite, essential oils, antibacterial activity, synergistic interaction, sustainable materials

## Abstract

The development of antimicrobial formulations for biodegradable wet wipes is of increasing interest for more sustainable hygiene applications. Objectives: This study aimed to develop and evaluate a cellulose-based antimicrobial wipe formulation by combining sodium hypochlorite with essential oils from *Cymbopogon citratus* (lemongrass), *Lavandula officinalis* (lavender), and *Syzygium aromaticum* (clove). The formulation was designed to incorporate sodium hypochlorite and essential oils into a cellulose matrix and to evaluate whether their combination could enhance antibacterial efficacy through synergistic interactions. Methods: Fourier-transform infrared spectroscopy with attenuated total reflectance (FTIR-ATR) was used as a complementary qualitative method to characterize the essential oils and to assess spectral changes following their incorporation into the cellulose-based wipe substrate. Antibacterial activity was evaluated against Gram-positive bacteria *Staphylococcus aureus* ATCC 25923 and *Staphylococcus epidermidis* ATCC 12228, and Gram-negative bacteria *Escherichia coli* ATCC 8739 and *Salmonella enteritidis* ATCC 13076 following CLSI guidelines, while combined interactions were assessed using the checkerboard assay. Results: FTIR-ATR analysis revealed characteristic spectral features of the investigated essential oils and detectable spectral changes following their incorporation into the sodium hypochlorite-treated cellulose-based wipes. Sodium hypochlorite exhibited the strongest antibacterial activity, while *S. aromaticum* oil showed the highest efficacy among essential oils. Notably, synergistic interactions were observed between sodium hypochlorite and all essential oils against Gram-positive bacteria, with lavender oil showing consistent synergy across all bacterial strains (FICI 0.28–0.31). The developed formulation achieved 99.999% bacterial reduction for Gram-positive strains and substantial inhibition of Gram-negative bacteria within 20 min. Conclusions: These findings demonstrate the potential of combining sodium hypochlorite with essential oils for the development of effective antibacterial cellulose-based wet wipes and provide a basis for further investigation of formulations that may achieve effective bacterial control with reduced concentrations of conventional disinfectants.

## 1. Introduction

The global demand for antibacterial wet wipes has increased substantially in recent years due to heightened awareness of hygiene and infection control practices. Despite their effectiveness in reducing microbial contamination, the environmental impact of these products has become a growing concern. Many commercially available wet wipes contain synthetic, nonwoven materials such as polyester and polypropylene, which are resistant to biodegradation and contribute to the accumulation of persistent waste and microplastics in natural ecosystems. These environmental challenges have driven research toward the development of sustainable alternatives that minimize ecological impact while maintaining antimicrobial performance [1,2].

Recent advances in material science have enabled the development of biodegradable nonwoven substrates derived from renewable resources such as cellulose, viscose, and cotton fibers. These materials offer improved environmental compatibility due to their ability to degrade under natural conditions, while still providing adequate mechanical strength and liquid retention properties required for hygiene applications. In this context, cellulose-based substrates are particularly promising because their renewable origin, biodegradability, absorbency, and fibrous structure make them suitable substrates for the incorporation of antimicrobial formulations. However, while progress in substrate design has been significant, the formulation of effective and environmentally friendly antimicrobial systems remains a critical area of investigation [3,4].

Concurrently, significant progress has been made in the exploration of natural antimicrobial agents, where essential oils have emerged as a major focus due to their broad-spectrum antimicrobial activity and natural origin. The antimicrobial mechanisms of essential oils have been extensively investigated and are primarily associated with disruption of microbial cell membranes, increased permeability, leakage of intracellular contents, and interference with enzymatic systems [5,6]. Recent study has also highlighted their potential synergistic interactions with conventional disinfectants, suggesting that combinations of natural and synthetic agents may enhance antimicrobial efficacy while reducing required dosages [7].

Among conventional disinfectants, sodium hypochlorite remains a benchmark agent due to its rapid, broad-spectrum antimicrobial activity against bacteria, viruses, and fungi. Its mode of action involves the generation of reactive chlorine species that induce oxidative damage to cellular components, including proteins, lipids, and nucleic acids [8,9,10]. Despite its effectiveness, concerns regarding irritation and tolerability at higher concentrations have prompted efforts to optimize its use in safer, lower-dose formulations [11].

Recent advances in formulation research have increasingly focused on the integration of sodium hypochlorite with naturally derived compounds, particularly essential oils, to achieve synergistic antimicrobial effects. Such combined systems have the potential to reduce the required concentrations of conventional disinfectants while preserving or enhancing overall antimicrobial efficacy [7,12]. The convergence of biodegradable substrates, plant-derived antimicrobial agents, and optimized disinfectant formulations represents a promising strategy for the development of next-generation wet wipes. Biodegradable substrates such as cellulose-based nonwovens not only ensure effective delivery of active agents but also facilitate environmentally responsible disposal through natural degradation processes. This integrated approach is consistent with current sustainability objectives and increasing regulatory demands aimed at minimizing plastic waste and reducing the environmental and health impacts of chemical agents in consumer products [13].

Despite increasing interest in biodegradable nonwoven substrates and plant-based antimicrobial systems, limited information is available on the combined use of sodium hypochlorite and essential oils in cellulose-based wet wipes, particularly regarding their antibacterial interactions and efficacy against representative Gram-positive and Gram-negative bacteria. In this context, the aim of this study was to develop cellulose-based wet wipes containing sodium hypochlorite and commercially available essential oils of *Lavandula officinalis* (lavender), *Cymbopogon citratus* (lemongrass), and *Syzygium aromaticum* (clove). The Fourier-transform infrared spectroscopy with attenuated total reflectance (FTIR-ATR) was employed as a complementary qualitative method to characterize the essential oils and to assess spectral changes associated with the incorporation of the active formulations into the cellulose-based wipes. The antibacterial activity of sodium hypochlorite and essential oils combinations was evaluated, with particular emphasis on identifying synergistic and additive interactions using checkerboard analysis. Furthermore, the optimized formulation was incorporated into cellulose-based wipes and evaluated for antibacterial efficacy against the Gram-positive bacteria *Staphylococcus aureus* ATCC 25923 and *Staphylococcus epidermidis* ATCC 12228 and the Gram-negative bacteria *Escherichia coli* ATCC 8739 and *Salmonella enteritidis* ATCC 13076.

## 2. Materials and Methods

### 2.1. Strains and Growth Conditions

Antibacterial activity of the essential oils and the disinfectant based on sodium hypochlorite (Aqualor H1000, Sigma, Crvenka, Serbia; pH 7.2, active chlorine 4 g·L^−1^) was tested against two Gram-positive bacteria *Staphylococcus aureus* ATCC 25923 and *Staphylococcus epidermidis* ATCC 12228 and the Gram-negative bacteria *Escherichia coli* ATCC 8739 and *Salmonella enteritidis* ATCC 13076. All bacterial strains were obtained from the Culture Collection of the microbiological laboratory at the Institute of Food Technology in Novi Sad (University of Novi Sad, Novi Sad, Serbia). Reference strains were preserved in Tryptone Soya Broth (TSB, Oxoid, Basingstoke, UK) supplemented with 40% glycerol at −80 °C and revitalized from frozen stocks by cultivation on tryptone soya agar (TSA, Oxoid, Basingstoke, UK) plates for 24 h at 37 °C before performing the assays.

### 2.2. Wipes Substrate

Cellulose-based wet wipes were prepared using commercially available cellulose sheets. The substrate material consists of a standardized, commercially available non-woven cellulose matrix derived from 100% plant-based fibers. The dry mass per unit area of the cellulose substrate was determined gravimetrically and was found to be 52 g·m^−2^. Cellulose, a natural polymer derived from plant sources, was chosen for its high absorbency, mechanical strength, and biodegradability. The cellulose sheets were cut into uniform dimensions of 10 × 10 mm to ensure consistent surface area for formulation and antimicrobial testing. Before testing, the wipes were exposed to ultraviolet (UV) radiation for 30 min to ensure surface sterilization.

### 2.3. Essential Oils

Commercially available essential oils were used as natural antimicrobial agents in the formulation of cellulose-based wet wipes solution. The selected oils included lavender oil (*L. officinalis*), lemongrass oil (*C. citratus*) and clove oil (*S. aromaticum)* were obtained from KIRKA CORPORATION DOO, Belgrade, Serbia. All experiments were conducted using essential oils from the same commercial batch to minimize variability during antimicrobial testing.

Due to the hydrophobic nature of essential oils, Tween 80 (0.5%, *v*/*v*) (Sigma-Aldrich, Germany) was used as a nonionic surfactant to improve their dispersion in TSB medium prior to antimicrobial testing. Control experiments containing the corresponding concentration of Tween 80 without essential oils were also included.

### 2.4. ATR–FTIR Spectroscopy Analysis

The chemical characterization of essential oils and wipes substrate was performed using an IRTracer-100 FTIR Spectrophotometer (Shimadzu, Japan) equipped with an attenuated total reflectance (ATR) module featuring a diamond crystal (Golden Gate™ High Temperature Heated Diamond ATR Top Plate, Specac, Orpington, UK).

A small volume of each essential oil sample was directly applied onto the ATR crystal without prior preparation. Spectra were recorded over the range of 4000–500 cm^−1^ at a resolution of 4 cm^−1^, with 32 scans collected per sample to improve the signal-to-noise ratio. Background spectra were obtained prior to each measurement under identical conditions and automatically subtracted from the sample spectra. FTIR-ATR analysis was performed as a complementary characterization method to confirm the presence of characteristic functional groups and evaluate the chemical consistency of the essential oils used in the study.

For the analysis of wipes, samples were pre-dried for 4 h at 60 °C (Memmert UNB 400, Memmert GmbH + Co. KG, Schwabach, Germany) to minimize interference from moisture and volatile constituents. Three different regions of each wipe were then analyzed under the same instrumental conditions as those applied for essential oils, including a spectral range of 4000–500 cm^−1^, a resolution of 4 cm^−1^, and the accumulation of 32 scans per sample. Background spectra were recorded prior to each measurement under identical conditions and automatically subtracted. The samples were placed directly onto the ATR crystal with adequate pressure to ensure optimal contact.

After each analysis, the ATR crystal was carefully cleaned with ethanol and dried to prevent cross-contamination. Spectral data were processed using the instrument’s software, and characteristic absorption bands were identified to determine the functional groups present in the essential oils.

#### FTIR-ATR Characterization of Cellulose-Based Wipes Impregnated with Sodium Hypochlorite and Natural Essential Oils

Cellulose-based wet wipes were previously dried at room temperature to remove residual moisture. After drying, each wipe was weighed, and the volume of the impregnation formulation was determined according to the dry mass of the substrate. Impregnation was performed using a ratio of 2.10 mL of formulation per gram of dry wipe mass, which was experimentally determined as the volume required for complete and homogeneous wetting of the entire substrate.

Disinfectant sodium hypochlorite (Aqualor H1000, Sigma, Crvenka, Serbia) was used for the preparation of the formulations, with the final concentration of active chlorine adjusted to 0.25 mg/mL. Essential oils of lavender, lemongrass, and clove were added individually or in a combination at concentration of 16.00, 2.00 and 1.00 mg/mL, respectively, based on the results obtained for the antibacterial activity of essential oils. In formulations intended for FTIR-ATR analysis, Tween 80 was added at a concentration of 0.5% *v*/*v* to improve the dispersion of hydrophobic essential oil components in the aqueous phase and to ensure more uniform impregnation of the cellulose substrate. The exact components and concentrations of the prepared treatment formulations are presented in Table 1.

The dried wipes were subsequently impregnated with the corresponding formulations in closed containers and left overnight to allow complete penetration of the formulations into the cellulose substrate. Following impregnation, the samples were dried for 2 h at 30 °C to reduce water content and improve the interpretability of the FTIR-ATR spectra while minimizing the loss of volatile essential oil components, and were subsequently reanalyzed by FTIR-ATR. FTIR-ATR spectra were recorded in the range of 4000–500 cm^−1^ at a resolution of 4 cm^−1^ using 32 scans per spectrum. A background spectrum was collected prior to sample analysis, and the ATR crystal was cleaned with ethanol between measurements. This approach enabled a clear interpretation of spectral changes and functional group interactions after water removal, reducing the baseline interference characteristic of wet samples.

### 2.5. Determination of Minimum Inhibitory Concentration (MIC) and Minimum Bactericidal Concentration (MBC)

The minimum inhibitory concentration (MIC) of sodium hypochlorite and essential oils was determined using a modified microdilution method in accordance with the Clinical and Laboratory Standards Institute guidelines M07-A10 [14]. Two-fold serial dilutions of the antimicrobial agents were prepared in Trypto-Casein Soy Broth (TSB; Biokar BK046 HA, Beauvais, France) in sterile 96-well microtiter plates, with a final concentration range from 0.25 mg/mL to 256 mg/mL. Due to the hydrophobic nature of essential oils, Tween 80 (0.5%, *v*/*v*) (Sigma-Aldrich, Darmstadt, Germany) was used as a nonionic surfactant to improve their dispersion in TSB medium prior to antimicrobial testing. Dilutions were prepared immediately before each experiment to ensure stability and homogeneous distribution of the essential oil and sodium hypochlorite. Subsequently, 100 μL of bacterial inoculum (1 × 10^6^ CFU/mL) was added to each well and the plates were incubated for 24 h at 37 °C. Positive controls (TSB medium containing bacterial inoculum without antimicrobial agents) and negative controls (TSB medium without inoculum) were included in all experiments. Control experiments containing the corresponding concentration of Tween 80 without essential oils were also included to exclude any antimicrobial effect of the surfactant itself. The tests were performed in four independent replicates. After incubation, 20 μL of a 0.01% resazurin solution (7-Hydroxy-3H-phenoxazin-3-one 10-oxide, HiMedia, Thane, India) was added to each well of the microtiter plate as an indicator of bacterial growth. Plates were further incubated at 37 °C for an additional 24 h. The MIC was defined as the lowest concentration of the antimicrobial agent that prevented a visible change in the color of resazurin from blue to pink, indicating inhibition of bacterial growth.

The minimum bactericidal concentration (MBC) of sodium hypochlorite and essential oils was determined following the MIC assay. From wells showing no visible bacterial growth in the MIC test, 10 µL aliquots were seeded in triplicate on Trypto-Casein Soy Agar (TSA; Biokar BK046 HA, Beauvais, France) plates and incubation for 24 h at 37 °C. MBC was defined as the lowest concentration of antimicrobial agent that resulted in complete bacterial inhibition, indicated by the absence of colony formation on agar plates.

### 2.6. Checkerboard Assay

The combined antimicrobial effect of sodium hypochlorite and essential oils was evaluated using a microdilution checkerboard assay [7,15]. Serial twofold dilutions of the tested agents were prepared in sterile 96-well microtiter plates. Sodium hypochlorite was diluted vertically to obtain final concentrations ranging from 1/16 × MIC to 4 × MIC, while essential oils were diluted horizontally across the plate within the same concentration range (1/16 × MIC to 4 × MIC). Following preparation of antimicrobial combinations, the bacterial suspension (1 × 10^6^ CFU/mL) prepared in TSB (Biokar BK046 HA, Beauvais, France) was added to each well and inoculated plates were incubated for 24 h at a temperature of 37 °C. Positive control (bacterial inoculum without antimicrobial agents) and negative control (TSB medium) were included in each experiment. The optical density (OD) was measured at 550 nm using a microplate reader (BioTek Epoch 2 Microplate Spectrophotometer, Agilent, Santa Clara, CA, USA). The test was performed in three independent replicates.

The fractional inhibitory concentration index (FICI) was calculated using the following equations:

FIC_A_ = MIC_AX_/MIC_AY_

FIC_B_ = MIC_BX_/MIC_BY_

FICI = FIC_A_ + FIC_B_

where FIC_A_ represents the fractional inhibitory concentration of the disinfectant sodium hypochlorite and FIC_B_ represents that of the essential oil. MIC_AX_ is the minimum inhibitory concentration (MIC) of sodium hypochlorite in the presence of the essential oil, while MIC_AY_ corresponds to the MIC of sodium hypochlorite alone. MIC_BX_ denotes the MIC of the essential oil in the presence of sodium hypochlorite, and MIC_BY_ is the MIC of the essential oil alone.

FICI values were interpreted according to the model proposed by Mulyaningsih et al., 2010 [15], as follows: synergistic effect (S), FICI ≤ 0.5; additive effect (Ad), 0.5 < FICI ≤ 1.0; indifferent effect (I), 1.0 < FICI ≤ 4.0; and antagonistic effect (A), FICI > 4.0.

### 2.7. Preparation of Antimicrobial Wet Wipes

The antimicrobial wet wipes used in the final antibacterial efficacy assay were prepared by impregnating sterile cellulose-based substrates with a freshly prepared combined antimicrobial formulation. The formulation contained sodium hypochlorite and three selected essential oils, namely lemongrass (*C. citratus*), lavender (*L. officinalis*), and clove (*S. aromaticum*). The final concentration of active chlorine was adjusted to 0.25 mg/mL, while the essential oils were incorporated at final concentrations of 2.00 mg/mL for *C. citratus*, 16.00 mg/mL for *L. officinalis*, and 1.00 mg/mL for *S. aromaticum*. Tween 80 was added at a concentration of 0.5% (*v*/*v*) to facilitate dispersion of the hydrophobic essential oils in the aqueous phase.

The components were combined and thoroughly mixed immediately before impregnation to obtain a homogeneous antimicrobial formulation. Sterilized cellulose substrates measuring 10 × 10 mm were individually placed in 12 well microtiter plates and impregnated with the freshly prepared formulation. A sufficient volume of the formulation was applied to completely wet the cellulose substrate and ensure uniform impregnation. Following impregnation, the 12 well microtiter plates were closed and the impregnated cellulose substrates were maintained at room temperature overnight to allow the formulation to penetrate and distribute throughout the cellulose matrix. The resulting impregnated cellulose substrates were used as antimicrobial wet wipes for further antibacterial efficacy assay.

### 2.8. Evaluation of the Antibacterial Activity of the Antimicrobial Formulation on Cellulose-Based Discs

The antibacterial efficacy of the prepared antimicrobial wet wipes was evaluated using a modified colony counting method on cellulose-based discs (10 × 10 mm), following the procedure described by Tomičić et al. 2023 [16]. Sterile cellulose discs were impregnated with the antimicrobial formulation described in Section 2.7 individually placed into the wells of a 12-well microtiter plate. Each disc was subsequently inoculated with 100 µL of bacterial suspension (1 × 10^6^ CFU/mL) and incubated at 21 °C for predetermined contact times of 1, 10 and 20 min. These time intervals were selected to provide standardized laboratory conditions for comparative assessment of antimicrobial interactions and formulation performance. After exposure, discs were transferred to 2 mL microcentrifuge tubes containing 1 mL of neutralization solution containing 1% Tween 80, 0.1% L-histidine and 0.5% Na-thiosulfate in TSB medium, selected based on previously reported neutralizing systems suitable for oxidizing disinfectants and essential oils containing formulations. Samples were centrifuged at 1500× *g* for 3 min. After neutralization, the number of viable cells was determined by standard colony counting technique on TSB (Biokar BK046 HA, Beauvais, France). Plates were then incubated for 24 h at 37 °C, after which colonies were counted. The test was performed in three independent replicates. Results were expressed as log CFU. A control dilution series prepared in TSB (Biokar BK046 HA, Beauvais, France) was included to verify the initial bacterial concentration. The antibacterial efficacy of the antimicrobial wet wipes (Ewet wipe) was calculated as the logarithmic reduction in bacterial counts on treated discs relative to the control and percentage reduction, according to the following equation:

E_wet wipe_ = LogNc − LogN_AS_

where

Nc represents the number of bacteria in the control suspension, and

N_AS_ represents the number of bacteria in the suspension after exposure to the antibacterial wet wipe solution.

## 3. Results

### 3.1. ATR-FTIR Spectrometry

The cellulose-based wipe sheets were homogeneous in appearance and composition, providing a consistent substrate for subsequent impregnation with sodium hypochlorite and essential oils.

**Figure 1:**
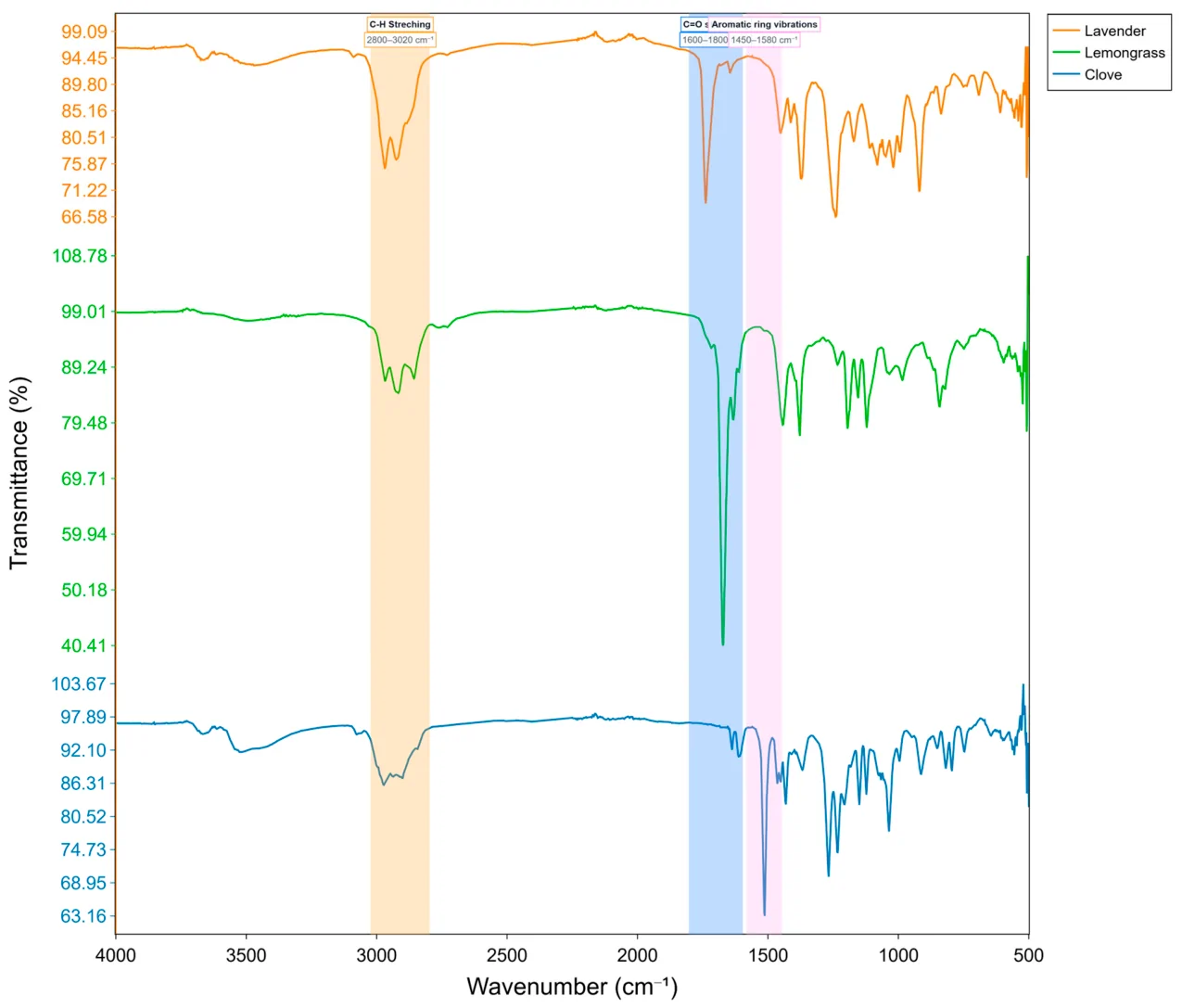
FTIR-ATR spectra of lavender essential oil, lemongrass essential oil, and clove essential oil. FTIR spectra were processed and publication-quality figures were generated using SpectraForge (Version 1.0, SpectraForge Software, FINS Institute, Novi Sad, Serbia).

The FTIR-ATR spectra of lavender, lemongrass, and clove essential oils (Figure 1) revealed both common absorption regions and distinct spectral features associated with differences in their predominant chemical composition. All three spectra showed characteristic absorptions in the 3000–2800 cm^−1^ region, corresponding to aliphatic C–H stretching vibrations. Although present in all investigated samples, this region showed only limited discriminatory potential.

More pronounced differences were observed in the carbonyl and unsaturated functional group region. Lavender exhibited a strong absorption band at approximately 1718 cm^−1^, consistent with C=O stretching vibrations associated with ester-containing constituents. In contrast, lemongrass showed its most intense absorption at approximately 1673 cm^−1^, accompanied by additional features in the adjacent region, reflecting the contribution of conjugated unsaturated oxygen-containing constituents characteristic of its chemical profile.

Clove was clearly differentiated from lavender and lemongrass by its characteristic absorptions in the aromatic ring vibration region. The dominant band at approximately 1512 cm^−1^, together with absorptions around 1600 cm^−1^, is consistent with aromatic C=C vibrations associated with its phenylpropanoid-rich composition. The differences observed in these characteristic spectral regions demonstrate the potential of FTIR-ATR spectroscopy for rapid discrimination among essential oils with distinct chemical profiles.

The FTIR-ATR spectrum of lemongrass essential oil showed a distinct spectral profile dominated by absorptions associated with oxygenated and unsaturated constituents. The characteristic C–H stretching vibrations were observed in the 3000–2850 cm^−1^ region, corresponding to aliphatic CH_3_ and CH_2_ groups. The most prominent absorption band, observed at approximately 1673 cm^−1^, can be attributed to C=O stretching vibrations of conjugated aldehyde structures, consistent with the presence of citral, one of the major constituents of lemongrass essential oil. Additional absorption bands in the 1630–1600 cm^−1^ region may be associated with C=C stretching vibrations of unsaturated structures. The complex fingerprint region, particularly between approximately 1250 and 1000 cm^−1^, contains several bands associated with C–O and C–C stretching vibrations of oxygenated terpenoid constituents.

FTIR-ATR spectroscopy was selected due to its rapid, non-destructive nature and minimal sample preparation, enabling efficient qualitative characterization based on functional group analysis. Although it does not provide detailed compositional data comparable to chromatographic techniques, it is highly suitable for rapid screening, differentiation, and confirmation of the main functional groups of essential oils through comparison with reference spectra and chemometric approaches.

**Figure 2:**
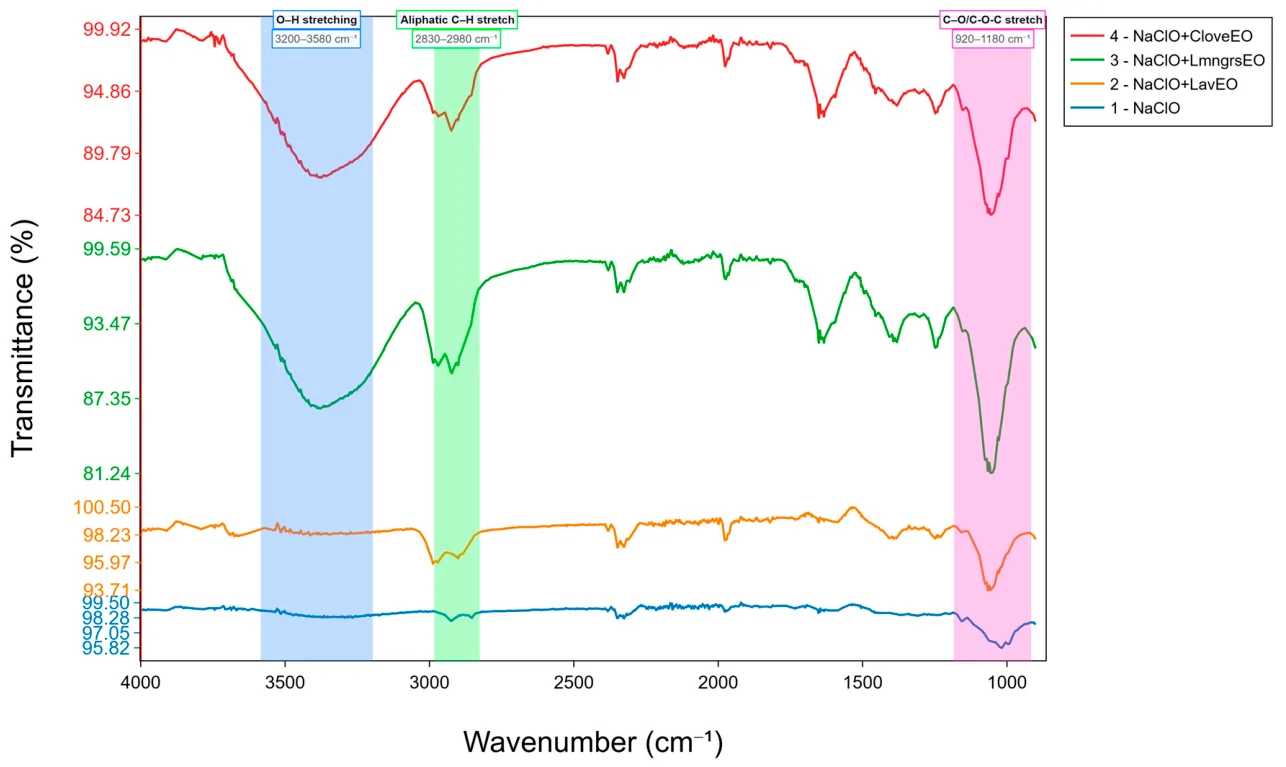
FTIR-ATR spectra of biodegradable cellulose-based wipes impregnated with sodium hypochlorite and essential oils. Spectra: (1) sodium hypochlorite; (2) sodium hypochlorite + lavender essential oil (EO); (3) sodium hypochlorite + lemongrass EO; and (4) sodium hypochlorite + clove EO. FTIR spectra were processed and publication-quality figures were generated using SpectraForge (Version 1.0, SpectraForge Software, FINS Institute, Novi Sad, Serbia).

FTIR-ATR analysis of wipes impregnated with NaClO and essential oils (Figure 2) revealed a common spectral profile dominated by the cellulose-based substrate, with additional spectral differences associated with the incorporation of lavender, lemongrass, and clove essential oils. A broad absorption band in the 3200–3580 cm^−1^ region, corresponding primarily to O–H stretching vibrations of the cellulose matrix and residual moisture, was clearly observed in the lemongrass- and clove-containing samples, whereas it was substantially attenuated and not clearly resolved in the NaClO and lavender-containing samples. The differences in the apparent intensity of this region should not be interpreted as a loss of cellulose hydroxyl groups, but rather as a possible consequence of differences in hydrogen-bonding interactions, surface hydration, and the local chemical environment of the cellulose matrix following impregnation. In addition, differences in the distribution and surface coverage of the essential-oil components may affect the relative contribution of the underlying cellulose signal in the ATR measurement.

The aliphatic C–H stretching region (2830–2980 cm^−1^) was more clearly expressed in the essential-oil-impregnated samples, particularly in comparison with the NaClO-treated sample, consistent with contributions from the organic constituents of the essential oils. The most pronounced differences among the samples were observed in the fingerprint region between approximately 920 and 1180 cm^−1^. This region contains several characteristic absorptions of the cellulose matrix, including C–O and C–O–C stretching vibrations, and may additionally contain overlapping contributions from oxygen-containing functional groups present in essential-oil constituents. The clove-containing sample exhibited the most pronounced spectral features in this region, while the lemongrass-containing sample also showed a complex absorption pattern. The lavender-containing sample displayed comparatively weaker changes, although characteristic absorptions remained evident in the lower-wavenumber region.

Additional differences were observed in the 1700–1400 cm^−1^ region, particularly for the lemongrass- and clove-containing samples. These changes may reflect contributions from functional groups associated with their major volatile constituents, including carbonyl, aromatic, and/or unsaturated structures, superimposed on the characteristic absorptions of the cellulose matrix. Overall, the observed spectral variations indicate that impregnation with different essential oils produces detectable changes in the FTIR-ATR profiles of the NaClO-treated wipes. The spectral profile therefore reflects the combined contribution of the cellulose substrate and the incorporated essential-oil components rather than a set of isolated essential-oil bands. However, the substantial overlap with the characteristic cellulose absorptions limits the unambiguous assignment of individual bands exclusively to the essential-oil components. Overall, the observed spectral variations indicate that impregnation with different essential oils produces detectable changes in the FTIR-ATR profiles of the NaClO-treated wipes. The spectral profile therefore reflects the combined contribution of the cellulose substrate and the incorporated essential-oil components rather than a set of isolated essential-oil bands. However, the substantial overlap with the characteristic cellulose absorptions limits the unambiguous assignment of individual bands exclusively to the essential-oil components.

### 3.2. Antibacterial Activity of Sodium Hypochlorite and Essential Oils

The antibacterial activity of disinfectant sodium hypochlorite and commercially available essential oils *C. citratus*, *L. officinalis* and *S. aromaticum* was evaluated against the reference strains of Gram-positive bacteria *S. aureus* ATCC 25923 and *S. epidermidis* ATCC 12228, and Gram-negative bacteria *E. coli* ATCC 8739 and *S. enteritidis* ATCC 13076. The obtained minimum inhibitory concentration (MIC) and minimum bactericidal concentration (MBC) values (Table 2) demonstrated that all tested bacterial strains showed susceptibility to the investigated antimicrobial agents. However, a markedly stronger antibacterial effect was observed for sodium hypochlorite compared to the essential oils, confirming its well-established efficacy as a potent disinfectant.

Among the tested essential oils, *S. aromaticum* exhibited the highest antibacterial activity, with MIC and MBC values ranging from 0.25 to 2.00 mg/mL across the tested strains. *C. citratus* also exhibited considerable antibacterial activity, although less potent than *S. aromaticum*. Its effectiveness was more pronounced against Gram-positive bacteria (MIC/MBC = 0.50–1.00 mg/mL) compared to Gram-negative strains, where significantly higher concentrations were required (4.00–8.00 mg/mL). In contrast, *L. officinalis* essential oil showed the weakest antimicrobial effect, with MIC and MBC values ranging from 4.00 to 256.00 mg/mL, indicating significantly lower potency. As expected, Gram-positive bacteria were more sensitive to the tested essential oils than Gram-negative bacteria.

### 3.3. Evaluation of Combined Antibacterial Effects of Sodium Hypochlorite and Essential Oils

Combination assays of sodium hypochlorite with essential oils of *C. citratus*, *L. officinalis*, and *S. aromaticum* were conducted using the microdilution checkerboard method to evaluate their joint antibacterial activity. Particular emphasis was placed on assessing these combinations as a basis for the development of optimized formulations for antimicrobial delivery system, in which essential oils could contribute to reducing the required levels of conventional antimicrobial agents. Interactions were quantitatively determined through fractional inhibitory concentration (FIC) and fractional inhibitory concentration index (FICI) values (Table 3).

The results demonstrated that the most tested combinations exhibited synergistic or additive effects, depending on the bacterial strain and the essential oil applied. Notably, all combinations of sodium hypochlorite with the investigated essential oils produced synergistic effect against Gram-positive bacteria, whereas in some cases interactions against Gram-negative strains were additive.

The combination of disinfectant with lavender essential oil exhibited consistent synergism across all tested reference strains, as indicated by FICI values ranging from 0.28 to 0.31. In contrast, sodium hypochlorite at subinhibitory concentrations from 1/4 to 1/16 MIC combined with clove essential oil at 1/4 MIC showed pronounced synergistic interactions against Gram-positive strains, while additive effect was observed against Gram-negative bacteria.

### 3.4. Antibacterial Efficacy of Cellulose-Based Wet Wipes Containing Sodium Hypochlorite and Essential Oils

Based on the observed interactions between sodium hypochlorite and the selected essential oils, a prototype formulation for antibacterial wipes was developed. The formulation was designed to exploit synergistic effects, as determined by fractional inhibitory concentration (FIC) in order to reduce the required levels of conventional antimicrobial agents. The final concentrations of sodium hypochlorite and essential oils were selected based on the overall optimization of antimicrobial efficacy, synergistic/additive interactions against all tested bacterial strains, and formulation stability within the cellulose-based wipe system. Although MIC and FIC values varied among bacterial strain, the selected concentrations represented the optimal compromise for achieving broad-spectrum antibacterial activity and reproducible performance in the final wet wipe formulation.

The antibacterial efficacy of the formulated wipe solution, containing sodium hypochlorite in the concentration of 0.25 mg/mL and essential oils from *C. citratus*, *L. officinalis*, and *S. aromaticum* in the concentrations 2.00, 16.00, 1.00 mg/mL, respectively, was evaluated on cellulose discs (10 × 10 mm) using the standard colony counting method on TSA. The results demonstrated that antibacterial activity increased with exposure time for all tested bacteria (Table 4). After 1 min of exposure, the weakest effect was observed against *S. enteritidis* ATCC 13076 (88.25%), whereas Gram-positive bacteria demonstrated higher susceptibility reaching up to 97.31% for *S. aureus* ATCC 25923. A significant increase in antibacterial activity was recorded after 10 min of exposure across all tested strains, with reduction percentage ranging from 99.82% to 99.99%. Notably, *S. aureus* ATCC 25923 remained the most susceptible bacteria (99.991%), while *E. coli* ATCC 8739 and *S enteritidis* ATCC 13076 showed lower susceptibility, exhibiting reductions of 99.86% and 99.82%, respectively. The highest antibacterial efficacy was achieved after 20 min of exposure, at which Gram-positive bacteria exhibited the greater susceptibility, exceeding 99.999%, while Gram-negative bacteria showed lower, but still considerable reduction. These results indicate that the combination of sodium hypochlorite with essential oils from *C. citratus*, *L. officinalis*, and *S. aromaticum* effectively inhibits both Gram-positive and Gram-negative bacteria on cellulose-based wet wipes, with efficacy strongly dependent on exposure time and bacterial strain.

## 4. Discussion

The increasing demand for sustainable and biodegradable hygiene products has driven research into eco-friendly alternatives to conventional wet wipes. In this study, we aimed to develop an antibacterial solution for cellulose-based wet wipes by combining sodium hypochlorite with commercially available essential oils of *L. officinalis*, *C. citratus* and *S. aromaticum*. The rationale behind this approach was to combine the strong disinfectant activity of sodium hypochlorite with the bioactive properties of essential oils and to investigate whether synergistic interactions could enhance its efficacy while potentially reducing the amount of sodium hypochlorite required for effective antibacterial activity. From a polymer-materials perspective, the study addresses the development of a biodegradable cellulose-based substrate capable of incorporating and delivering multiple antimicrobial agents, thereby combining the intrinsic functionality of a renewable polymer with the activity of bioactive compounds. From a polymer chemistry perspective, cellulose matrix represents a highly suitable matrix for antimicrobial formulations because of its linear β-(1→4)-D-glucan structure, which contains abundant hydroxyl groups capable of forming an extensive hydrogen-bonding network [17,18]. These hydroxyl functionalities impart high hydrophilicity and water absorbency while providing numerous interaction sites for formulation components. Sodium hypochlorite, present in aqueous solution predominantly as hypochlorous acid (HOCl) and hypochlorite ions (OCl^−^) depending on pH, can be readily distributed throughout the hydrated cellulose network. In contrast, the major essential oil constituents, eugenol (phenolic aromatic compound), citral (α,β-unsaturated aldehyde), and linalyl acetate (terpenoid ester), are relatively hydrophobic molecules [19,20,21,22]. Their homogeneous distribution within the aqueous phase was facilitated by Tween 80, whereas their retention within the cellulose matrix is expected to occur primarily through physical adsorption, hydrogen bonding involving oxygen-containing functional groups, and hydrophobic interactions rather than covalent bonding. The cellulose substrate therefore acts not only as a biodegradable carrier but also as a functional polymeric platform that enables absorption, retention, and delivery of antimicrobial components. The interaction between polymer structure and active compounds is an important factor determining the performance of the final antimicrobial material [18,23].

Overall, FTIR-ATR analysis provided complementary evidence of spectral changes associated with the incorporation of essential oils into the sodium hypochlorite-treated cellulose-based wipes. The observed differences between the impregnated formulations were consistent with the presence of additional organic constituents, although substantial overlap with the cellulose-related absorptions limited the unambiguous assignment of individual bands exclusively to the essential oils. Thus, FTIR-ATR was used primarily as a complementary qualitative tool to characterize the essential oils and assess spectral changes following their incorporation into the wipe substrate, rather than for detailed structural characterization [20,24,25,26,27].

Taken together, the FTIR-ATR, antibacterial, and synergy results indicate that the performance of the cellulose-based wipe cannot be attributed solely to the intrinsic antibacterial activity of the individual essential oils. The cellulose matrix itself provides a chemically active environment characterized by abundant hydroxyl groups and glyosidic C–O–C linkages, which are responsible for its characteristic O–H, C–H, and fingerprint-region absorptions. In particular, the 1160–1030 and approximately 900 cm^−1^ regions contain vibrations associated with the β-(1→4)-glycosidic structure and C–O/C–O–C bonds of cellulose [28]. Citral contains an α,β-unsaturated aldehyde functionality, with characteristic C=O and C=C absorptions reported in the approximately 1670 and 1600–1630 cm^−1^ regions [29]. Linalyl acetate contains an ester group and unsaturated bonds, with characteristic es-ter C=O, C–O, and C=C absorptions reported around 1735, 1237–1250, and 1630 cm^−1^, respectively [4]. These structural differences provide a plausible chemical basis for the distinct FTIR profiles observed for the essential-oil-containing wipes.

**Figure 3:**
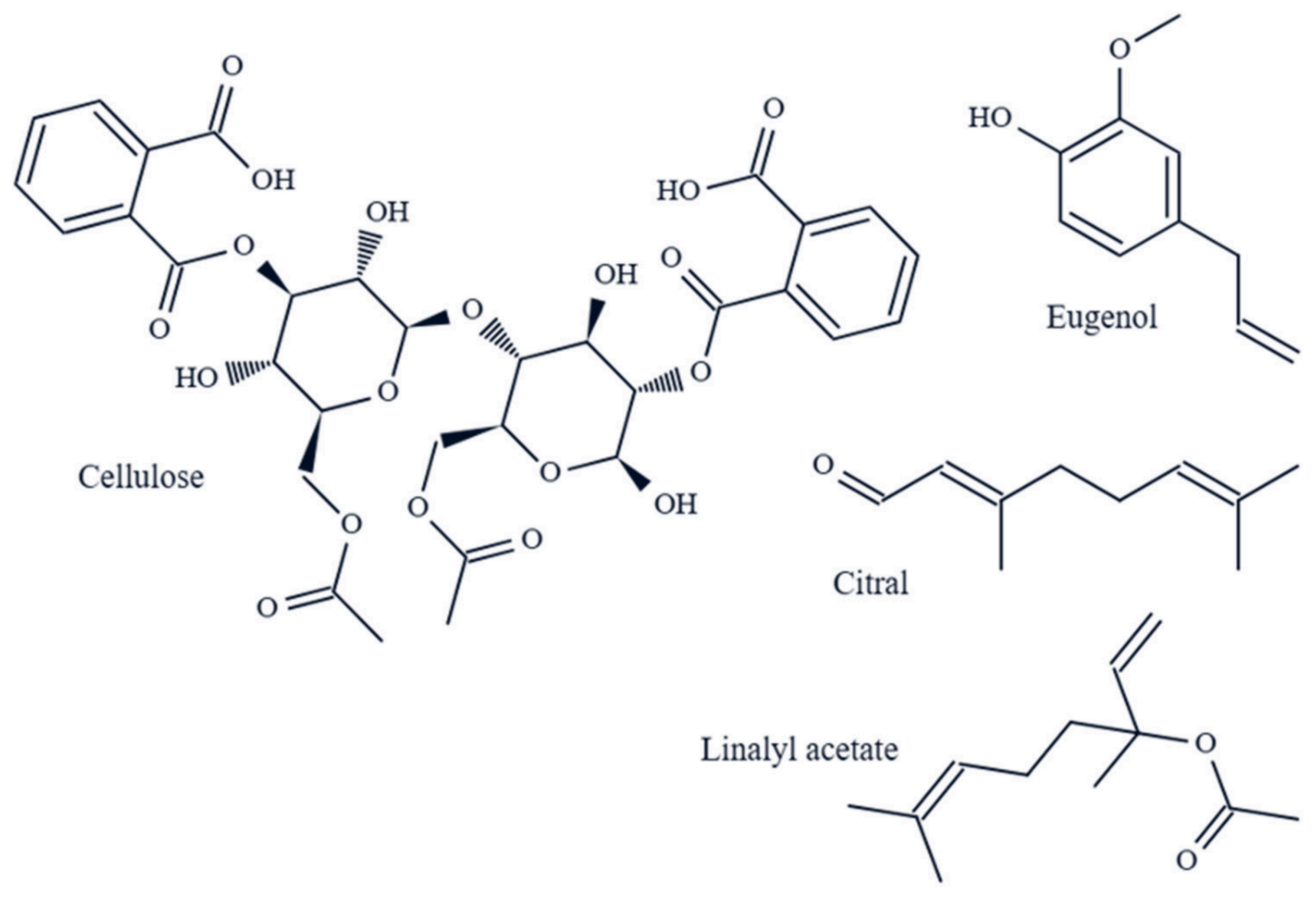
Chemical structures of cellulose and representative essential-oil constituents corresponding to the highest FTIR library matches: eugenol, citral, and linalyl acetate.

The observed spectral differences can be further interpreted by considering the chemical structures of the cellulose matrix and representative constituents of the essential oils (Figure 3). Cellulose is a linear β-(1→4)-linked polysaccharide containing numerous hydroxyl groups and glycosidic C–O–C linkages, which contribute substantially to its characteristic O–H, C–H and C–O–C absorptions in the FTIR spectrum. The broad O–H stretching region is particularly sensitive to the hydrogen-bonding environment and interactions with water, whereas the fingerprint region contains characteristic vibrations associated with the glycosidic structure of cellulose [30].

In contrast, the representative essential-oil constituents corresponding to the highest FTIR library matches possess structurally distinct functional groups that may contribute to the additional spectral features observed after impregnation. Eugenol, corresponding to the highest library match for the clove essential-oil-containing sample (92.0%), is a phenolic compound containing a hydroxyl group, a methoxy group, an aromatic ring and an allyl substituent. These functionalities are consistent with re-ported FTIR features of eugenol, including O–H, C=C and C–O–C-related absorptions [31]. Citral, corresponding to the highest library match for the lemongrass essential oil containing sample (94.6%), contains an α,β-unsaturated aldehyde functionality and conjugated carbon–carbon double bonds, with characteristic FTIR bands reported for C=O and C=C stretching [32]. Linalyl acetate, corresponding to the highest library match for the lavender essential oil containing sample (88.3%), contains an ester group and carbon–carbon double bonds, which are associated with characteristic C=O, C–O and C=C absorptions in FTIR spectra [33].

These structural differences provide a plausible chemical basis for the spectral variations observed among the essential-oil-containing wipes, particularly in the 1700–1400 and 1180–920 cm^−1^ regions. Nevertheless, the extensive overlap between cellulose and essential-oil-related absorptions prevents unequivocal assignment of individual bands to specific essential-oil constituents based on FTIR-ATR alone.

The coexistence of cellulose, sodium hypochlorite, and essential-oil constituents may also influence the local chemical environment of the impregnated wipe. The hydroxyl groups of cellulose can participate in hydrogen-bonding interactions with oxygen-containing functionalities of organic molecules, potentially modifying the local hydrogen-bonding environment and the accessibility of functional groups at the cellulose surface. FTIR spectroscopy is particularly sensitive to such changes in cellulose hydroxyl interactions and hydration [30]. Furthermore, sodium hypochlorite is capable of promoting oxidative modification of cellulose hydroxyl groups under suitable reaction conditions, as demonstrated for hypochlorite-based oxidation systems [31]. This intricate chemical environment directly influences the retention and synergistic antibacterial performance of the active agents based on their functional groups. Eugenol contains a polar phenolic hydroxyl group that forms strong hydrogen bonds with the cellulose matrix, which correlates with the distinct spectral shifts in the O–H stretching and fingerprint regions. This strong polymeric anchoring prevents rapid volatilization, providing a sustained and highly stable synergistic effect when combined with the rapid oxidative action of sodium hypochlorite. Conversely, citral (an aldehyde) and linalyl acetate (an ester) lack free hydroxyl groups, interacting with the cellulose backbone primarily through weaker dipole–dipole or hydrophobic interactions, as evidenced by the localized C=O and C=C stretching bands in the 1700–1400 cm^−1^ region. Consequently, these components exhibit faster desorption kinetics, yielding a rapid initial burst of antibacterial activity but a more transient synergistic interaction with sodium hypochlorite [34]. Therefore, oxidative modification of susceptible functional groups cannot be excluded as a possible contributor to the observed spectral differences. However, the present FTIR-ATR data do not provide direct evidence for specific oxidation products or reaction pathways, and these processes should consequently be regarded as possible mechanisms rather than demonstrated chemical transformations.

The antibacterial results clearly demonstrated the superior efficacy of sodium hypochlorite compared to the tested essential oils, confirming its role as a highly potent, broad-spectrum antimicrobial agent. Its pronounced activity can be attributed to its strong oxidative capacity, which leads to rapid and irreversible damage to key cellular components, including proteins, lipids, and nucleic acids [35,36]. Nevertheless, the essential oils exhibited distinct, compound-dependent antibacterial effects. Among them, *S. aromaticum* showed the highest activity, which is consistent with its typically high eugenol content. As a phenolic compound, eugenol exerts antimicrobial activity primarily through disruption of cell membrane integrity, increased permeability, and interference with enzymatic and metabolic processes, ultimately leading to leakage of intracellular constituents and eventual cell death [37,38,39].

In contrast, *L. officinalis* essential oil demonstrated comparatively weaker antibacterial activity, which is likely associated with its chemical composition dominated by esters such as linalyl acetate and other monoterpenes. Multiple studies have shown that the antibacterial efficacy of lavender essential oil varies with its chemical profile, with esters generally exhibiting milder activity compared to phenolic compounds due to lower membrane-disrupting potential [40,41]. This marked difference in activity highlights the critical influence of chemical structure on antimicrobial potency and underscores the importance of selecting essential oils based on the nature and relative abundance of their major bioactive components when designing effective antimicrobial formulations. For the cellulose-based material, these differences are additionally important because the chemical structure of the active compounds may influence not only their intrinsic antimicrobial activity but also their affinity for the hydrophilic polymer matrix and, consequently, their availability at the material–microorganism interface.

The observed higher susceptibility of Gram-positive bacteria compared to Gram-negative bacteria is in agreement with established knowledge regarding bacterial cell wall structure. The outer membrane of Gram-negative bacteria acts as a permeability barrier, limiting the penetration of hydrophobic compounds such as essential oils and other antimicrobial agents [42,43]. In contrast, the relatively simpler cell wall of Gram-positive bacteria lacks this additional barrier, facilitating greater accessibility of active compounds to the cytoplasmic membrane and internal targets [44,45].

Importantly, the combination assays revealed predominantly synergistic and, in some cases, additive interactions between sodium hypochlorite and the tested essential oils. These findings are particularly significant in the context of the escalating global threat of antimicrobial resistance (AMR) and indicate that carefully optimized combinations may achieve effective antimicrobial activity while potentially reducing the required concentration of conventional disinfectants. The widespread and often indiscriminate use of conventional disinfectants and antibiotics has accelerated the emergence of resistant microbial populations, underscoring the urgent need for alternative antimicrobial strategies that maintain efficacy while limiting the development of resistance. In this regard, essential oils represent promising candidates due to their chemically complex nature and multi-target modes of action [16,46].

Furthermore, while previous studies investigating sodium hypochlorite and essential oil combinations mainly reported additive or indifferent effects [7], the present study demonstrated synergistic interactions for selected formulations, highlighting the potential of carefully optimized combinations to improve antimicrobial performance while reducing the required concentration of sodium hypochlorite. Such optimization may be relevant for the development of cellulose-based antimicrobial wipes in which the amount of conventional disinfectant can potentially be reduced without compromising antibacterial performance. Moreover, the application of these optimized formulations directly onto biodegradable wipe substrates extends the practical relevance of the study and represents a still limited area of research in antimicrobial material development.

The observed synergistic interactions, particularly those involving lavender essential oil, suggest a mechanistic basis rooted in membrane-level effects. Essential oil constituents are known to perturb lipid bilayers, increasing membrane fluidity and permeability, which may facilitate enhanced intracellular penetration of sodium hypochlorite. This cooperative mechanism likely potentiates oxidative damage within the cell, leading to more efficient microbial inactivation [47]. Consequently, such synergistic interactions suggest the possibility of reducing concentrations of conventional disinfectants while maintaining antimicrobial performance, thereby supporting the development of more sustainable and resistance-conscious formulations. Recent studies have further emphasized that combination-based antimicrobial strategies may contribute to more prudent antimicrobial use by reducing required concentrations of active agents while maintaining efficacy [7,20,27].

The practical applicability of the proposed formulation was further substantiated through its implementation on cellulose-based wet wipes, where a pronounced time-dependent antibacterial effect was observed. This kinetic behaviour suggests a cumulative antimicrobial action associated with sustained interaction between active agents and microbial cells. Notably, the high level of antibacterial activity 99.999% achieved after 20 min confirms that the developed system is effective against Gram-positive bacteria and Gram-negative bacteria. Importantly, measurable antibacterial activity was also observed at the shortest tested exposure time, indicating the potential suitability of the developed formulations for practical disinfection applications. Nevertheless, further studies under shorter, real-use contact conditions are necessary to fully assess their effectiveness during typical consumer use.

The results demonstrate that cellulose matrix can function as a biodegradable polymeric platform for the incorporation and delivery of a multi-component antimicrobial system. The combination of a renewable cellulose matrix, sodium hypochlorite, and essential oils provides an approach in which polymer structure, formulation chemistry, and antimicrobial mechanisms are integrated within a single functional material. The observed synergistic interactions are particularly relevant because they indicate that antimicrobial performance may be enhanced through formulation design rather than by increasing the total loading of conventional disinfectants. This structure–composition–performance relationship is of direct relevance to the development of multifunctional cellulose-based materials and supports further investigation of cellulose matrix as a platform for sustainable antimicrobial products.

## 5. Conclusions

In conclusion, to the best of our knowledge this is the first study to investigate sodium hypochlorite–essential oil combinations specifically for biodegradable cellulose-based wet wipes, integrating synergistic interaction analysis, FTIR-ATR characterization of the impregnated substrate, and evaluation of antibacterial performance in the final wipe format. The results confirmed that although sodium hypochlorite remains the primary contributor to antimicrobial activity, the incorporation of essential oils can contribute to enhanced antibacterial activity through synergistic or additive interactions, depending on the essential oil and bacterial strain. These effects are strongly influenced by the chemical composition of the oils, highlighting the importance of selecting bioactive compounds with potent antimicrobial mechanisms. From a polymer-materials perspective, the findings demonstrate that cellulose matrix can function as a biodegradable and highly absorbent polymeric platform for the physical incorporation and delivery of chemically diverse antimicrobial agents.

The observed synergism between sodium hypochlorite and essential oils is especially significant, as it indicates the potential to reduce the concentration of conventional disinfectants required to achieve effective antibacterial activity. Such combination-based strategies may provide a basis for developing more efficient antimicrobial formulations in which the activity of conventional disinfectants is complemented by naturally derived antimicrobial compounds. Importantly, this approach enables the antimicrobial functionality of the cellulose substrate to be tuned through formulation composition without requiring permanent chemical modification of the cellulose backbone. The resulting material therefore combines the intrinsic physicochemical advantages of cellulose matrix with the functional activity of incorporated antimicrobial compounds.

By combining renewable cellulose-based polymeric substrate with naturally derived bioactive compounds and conventional disinfectants, this study provides a promising foundation for developing functional biodegradable antimicrobial wipes. The results demonstrate the potential of cellulose matrix to serve as a functional carrier for multi-component antimicrobial formulations while retaining its characteristic cellulose chemical profile. Moreover, the findings provide a basis for the design of next-generation cellulose-based wet wipes with potentially improved antimicrobial efficiency, and they encourage further research into optimizing such synergistic antimicrobial systems under shorter contact times, storage conditions, and practical use scenarios. Future research should focus on optimizing the formulation and polymer–active-agent interactions to achieve controlled loading, stability, rapid but effective antimicrobial action, and preservation of the mechanical and biodegradation characteristics of the cellulose substrate. Studies under shorter contact times, realistic storage conditions, repeated-use scenarios, and relevant biodegradation environments will be particularly important for translating the developed material from laboratory-scale proof of concept to practical sustainable antimicrobial products.

## Figures and Tables

**Table 1:** Detailed composition and concentrations of the experimental treatment formulations used for wipe impregnation.

Formulation	Active Chlorine (mg/mL)	Essential Oil Type	Concentration (mg/mL)	Surfactant (Tween 80, *v*/*v*)
NaClO	0.25	None	-	0.50%
Lavender EO	0.25	Lavender (*L. officinalis*)	16.00	0.50%
Lemongrass EO	0.25	Lemongrass (*C. citratus*)	2.00	0.50%
Clove EO	0.25	Clove (*S. aromaticum*)	1.00	0.50%

**Table 2:** Antimicrobial activity of the agents.

	Gram-Positive Bacteria				Gram-Negative Bacteria			
	*Staphylococcus* *aureus* ATCC 25923		*Staphylococcus epidermidis* ATCC 12228		*Escherichia coli* ATCC 8739		*Salmonella enteritidis* ATCC 13076	
Antimicrobial agent:	MIC (mg/mL)	MBC (mg/mL)	MIC (mg/mL)	MBC (mg/mL)	MIC (mg/mL)	MBC (mg/mL)	MIC (mg/mL)	MBC (mg/mL)
Sodium hypochlorite	0.25 ± 0.00	0.25 ± 0.00	0.25 ± 0.00	0.25 ± 0.00	0.50 ± 0.00	0.50 ± 0.00	0.50 ± 0.00	0.50 ± 0.00
*Cymbopogon citratus*	0.50 ± 0.00	0.50 ± 0.00	1.00 ± 0.00	1.00 ± 0.00	4.00 ± 1.00	4.00 ± 0.00	8.00 ± 2.00	8.00 ± 0.00
*Lavandula officinalis*	4.00 ± 0.00	8.00 ± 2.00	4.00 ± 0.00	4.00 ± 0.00	8.00 ± 2.00	8.00 ± 0.00	256.00 ± 64.00	256.00 ± 0.00
*Syzygium aromaticum*	0.25 ± 0.00	0.25 ± 0.00	0.50 ± 0.00	0.25 ± 0.00	1.00 ± 0.00	1.00 ± 0.00	2.00 ± 0.00	2.00 ± 0.00

Values represent the mean value ± SD obtained in four independent replicates. MIC—minimum inhibitory concentration, MBC—minimum bactericidal concentration.

**Table 3:** Interaction effects of combinations of sodium hypochlorite with essential oils.

Bacteria	MIC [mg·mL^−1^]		FIC		FICI	Interaction Effect
	Disinfectant	Essential Oil	Disinfectant	Essential Oil		
Combination of disinfectant with *Cymbopogon citratus* essential oil						
*Staphylococcus aureus* ATCC 25923	0.06 ± 0.00	0.13 ± 0.00	0.25 ± 0.07	0.25 ± 0.00	0.50 ± 0.07	S
*Staphylococcus epidermidis* ATCC 12228	0.03 ± 0.01	0.25 ± 0.07	0.13 ± 0.00	0.25 ± 0.07	0.38 ± 0.07	S
*Escherichia coli* ATCC 8739	0.06 ± 0.02	2.00 ± 0.57	0.13 ± 0.04	0.50 ± 0.14	0.63 ± 0.18	Ad
*Salmonella enteritidis* ATCC 13076	0.03 ± 0.01	4.00 ± 1.15	0.06 ± 0.02	0.50 ± 0.14	0.56 ± 0.16	Ad
Combination of disinfectant with *Lavandula officinalis* essential oil						
*Staphylococcus aureus* ATCC 25923	0.02 ± 0.00	2.00 ± 0.57	0.06 ± 0.02	0.25± 0.07	0.31 ± 0.09	S
*Staphylococcus epidermidis* ATCC 12228	0.02 ± 0.00	1.00 ± 0.29	0.06 ± 0.02	0.25 ± 0.07	0.31 ± 0.09	S
*Escherichia coli* ATCC 8739	0.06 ± 0.02	2.00 ± 0.00	0.13 ± 0.00	0.25 ± 0.07	0.38 ± 0.07	S
*Salmonella enteritidis* ATCC 13076	0.02 ± 0.00	64.00 ± 0.00	0.03 ± 0.00	0.25 ± 0.07	0.28 ± 0.07	S
Combination of disinfectant with *Syzygium aromaticum* essential oil						
*Staphylococcus aureus* ATCC 25923	0.02 ± 0.00	0.06 ± 0.02	0.06 ± 0.00	0.25 ± 0.07	0.31 ± 0.07	S
*Staphylococcus epidermidis* ATCC 12228	0.06 ± 0.02	0.13 ± 0.04	0.25 ± 0.07	0.25 ± 0.07	0.50 ± 0.14	S
*Escherichia coli* ATCC 8739	0.13 ± 0.04	1.00 ± 0.29	0.25 ± 0.07	0.50 ± 0.00	0.75 ± 0.07	Ad
*Salmonella enteritidis* ATCC 13076	0.13 ± 0.00	1.00 ± 0.29	0.25 ± 0.00	0.50 ± 0.14	0.75 ± 0.14	Ad

Values represent the mean values ± SD obtained in three independent replicates. MIC—minimum inhibitory concentration, FIC—fractional inhibitory concentration, FICI—fractional inhibitory concentrations index. Interaction effect: S—synergistic, Ad—additive.

**Table 4:** Antibacterial efficacy of the wet wipe formulation at different exposure times (1, 10, and 20 min) expressed as logarithmic reduction (log CFU·mL^−1^).

Bacteria	N_c_ [log CFU·mL^−1^]	Exposure Time 1 min	Exposure Time 10 min	Exposure Time 20 min
		E_wet wipe_ [Log CFU·mL^−1^]	E_wet wipe_ [Log CFU·mL^−1^]	E_wet wipe_ [Log CFU·mL^−1^]
*Staphylococcus aureus* ATCC 25923	7.04 ± 0.11	1.57 ± 0.03	4.04 ± 0.08	6.74 ± 0.02
*Staphylococcus epidermidis* ATCC 12228	7.11 ± 0.15	1.46 ± 0.04	3.93 ± 0.03	6.63 ± 0.01
*Escherichia coli* ATCC 8739	6.85 ± 0.09	0.95 ± 0.06	2.85 ± 0.05	5.59 ± 0.04
*Salmonella enteritidis* ATCC 13076	6.93 ± 0.10	0.93 ± 0.01	2.75 ± 0.01	5.40 ± 0.02

Values represent the mean values ± SD obtained in three independent replicates. N_c_—initial number of bacteria in the control suspension; E_wet wipe_—antibacterial efficacy of the wet wipe solution.

## Data Availability

The original contributions presented in this study are included in the article. Further inquiries can be directed to the corresponding author.
